# Breastfeeding and overweight/obesity among children and adolescents: a cross-sectional study

**DOI:** 10.1186/s12887-022-03394-z

**Published:** 2022-06-16

**Authors:** Fange Liu, Di Lv, Lumin Wang, Xiaoyu Feng, Rongjun Zhang, Wendong Liu, Wenchao Han

**Affiliations:** 1 Pediatric Nephrology and Endocrinology Department, the First Affiliated Hospital of Shandong First Medical University &Shandong Provincial Qianfoshan Hospital, No.16766 Jingshi Road, Lixia District, Jinan, 250014 China; 2 Department of Pediatrics, Qingdao Municipal Hospital, Affiliated to Qingdao University, Qingdao, 266071 China; 3 Qingdao Medical College, Qingdao University, Qingdao, 266071 China; 4 Department of Pediatrics, Qilu Hospital of Shandong University, Qingdao, 266035 Shandong China

**Keywords:** Children and adolescents, Breastfeeding, BMI, Overweight and obesity

## Abstract

**Background:**

Overweight and obesity are major public health crises among children and adolescents and contribute to a significant economic burden. We aimed to investigate the relationship between breastfeeding duration and overweight and obesity in children and adolescents in Qingdao, China in 2017.

**Methods:**

This study conducted a survey with 10,753 students (5370 boys and 5383 girls) aged 6 to 16 years from the Shibei District of Qingdao, China in 2017. Anthropometric measurements were taken by well-trained personnel and self-completed questionnaires were used to collect data from students. A variety of statistical methods were used in this study, including univariate and multivariate analyses, as well as linear and nonlinear regression models.

**Results:**

The prevalence of overweight and obesity was 15.45% and 19.76%, respectively. There was a significant negative correlation between breastfeeding duration and BMI in children and adolescents (β = -0.025, 95% CI: -0.033, -0.005, *P* < 0.01). Among boys, the BMI in children and adolescences of those who have been breastfed for more than 12 months was significantly lower than that of others whose breastfeeding duration was less than 12 months (β = -0.440, 95%CI -0.655, -0.224, *P* < 0.01). Breastfeeding has a particularly positive effect on the prevalence of obesity in boys aged 9 to 11 years (OR = 0.978, 95% CI: 0.958,0.999, *P* < 0.05).

**Conclusion:**

Breastfeeding can significantly reduce the prevalence of overweight and obesity among children and adolescents aged 6 to 16 years. Those who were breastfed for more than 12 months had a lower risk of developing overweight and obesity, especially boys between the ages of 9 and 11.

**Supplementary Information:**

The online version contains supplementary material available at 10.1186/s12887-022-03394-z.

## Introduction

In recent decades, obesity in children and adolescents has emerged as a serious health issue worldwide, including in Asian countries [1]. From 1990 to 2015, the prevalence of obesity doubled in more than 70 countries around the world [2, 3]. China has had the largest number of obese people in the world since 2016, and the childhood obesity rate is also rising [4]. The first Report on Childhood Obesity in China showed that from 1985 to 2014, the prevalence of overweight among children and adolescents aged 7-18 years increased sixfold (2.1% to 12.2%), while the obesity prevalence increased nearly fifteen times (0.5% to 7.3%). Without effective intervention, it is estimated that by 2030, the number of overweight and obese children and adolescents in China will reach 49.48 million [5]. Childhood obesity is caused by the occurrence of many disorders such as lipid disorders, increased glucose levels, abnormal blood pressure, insulin resistance and exercise intolerance. They all lead to the development of many chronic diseases during adulthood, including cardiovascular diseases, hypertension, type 2 diabetes and metabolic syndrome [6–8].

Various factors are involved in the prevalence of obesity, including genetic implications, birth weight, parental obesity, physical activity levels, socioeconomic status, age, sex, etc [9]. A higher protein intake early in life has been linked to body type in later life as it increases the secretion of insulin-like growth factor-1, which may stimulate growth and adiposity [10]. The various nutrients contained in breast milk are the most suitable nutrients for digestion and absorption in infants, and have the highest bioavailability. These nutrients can meet the physiological needs of infants in different developmental stages and cannot be replaced by any other food [11]. Over the previous decade, many studies have indicated an association between breastfeeding and childhood obesity. In a large birth cohort of 42,550 children from Southeast China, Zheng et al. found that longer duration of exclusive breastfeeding (EBF) was associated with lower risk of overweight [12]. A meta-analysis of 39 studies published by the World Health Organization (WHO) also showed that prolonging the duration of breastfeeding reduces the risk of obesity in children [13], while some previous studies showed no significant association between breastfeeding and childhood overweight and obesity in American and European samples [14, 15]. The question of whether breastfeeding protects children and adolescents from obesity is still debated, despite a large number of related publications.

As a representative emerging city in China, Qingdao has experienced rapid economic and social development in recent years. Meanwhile, the residents’ food consumption patterns have shifted from grain-based pattern to a high-protein and high-fat pattern including foods such as meat, aquatic products, and fried food [16, 17]. Subsequently, the prevalence of overweight and obesity among children and adolescents in Qingdao, especially those living in urban areas, has increased significantly in recent years [18]. However, there are few studies on the influencing factors of prevalence of overweight and obesity among children and adolescents in China's urban areas. Therefore, this study aimed to explore the relationship between breastfeeding and obesity among children and adolescents in the Shibei District of Qingdao which is an emerging first-tier coastal city in China.

## Materials and method

### Participants

Our study was based on the routine health examination project with primary and secondary school students from the Shibei District of Qingdao in 2017. This project was carried out under the guidance of the Qingdao Municipal Center for Disease Control and Prevention [2017] No. 29 document. The sample size was estimated according to the sample size calculation formula of the cross-sectional study, where α=0.05, ε=0.05, and p was the expected prevalence rate. The expected prevalence rates of overweight and obesity in children and adolescents were 12.2% and 7.3% respectively [5], so the calculated sample size was 11,059 students. We surveyed 11,731 students totally. After we excluded 978 low-weight students, 10,753 students participated in our statistical analysis. Informed consent was obtained from their parents.

### Measurement

The research team was composed of a professional medical team from Qilu Hospital (Qingdao) of Shandong University and personnel from the education bureau personnel division in the Shibei District of Qingdao. Only doctors and nurses who have received standardized training were qualified to take the students’ physical measurements. The physical measurements in this study mainly focused on the students’ weights and heights. Body height (standing height without shoes) was measured to the nearest 0.1 cm using a Harpenden stadiometer (Holtain Ltd., Crymych, UK) and body weight was measured to the nearest 0.1 kg with a calibrated scale (Seca, Hamburg, Germany) with each child wearing only underwear. Each measurement was repeated, and the average was used for the analyses (a third measurement was obtained if the first two measurements were more than 0.5 cm or 0.5 kg apart, and the average of the two closest measurements was used in the analyses). All measuring instruments were calibrated by standard before use, and the error among all measuring instruments was less than 10%. Body mass index (BMI) was calculated as follows: weight (kg) / height (m) ^2^.

### Questionnaire

Personnel from the education bureau personnel division were responsible for gathering information from the questionnaires. Questionnaires completed by the students and their parents were used to collect the demographic information of family members, the children’s birth conditions, feeding conditions and growth and development conditions, etc. The questionnaire mainly centered on the following aspects: parents’ age, height (cm), ethnicity, parity, birth gestational week, mode of delivery and students’ age, sex, birth length (cm), birth weight (kg), breastfeeding duration (months), supplementary feeding time (month), teething time (month), vision, number of cavities, etc.

### Overweight and obesity

In this study, obesity and overweight in different age groups and in both sexes were defined according to the standards for obesity in the Screening for overweight and obesity among school-age children and adolescents released in 2018 by the National Health and Family Planning Commission, People’s Republic of China.

### Covariates

The following sociodemographic and exposure factors derived from the questionnaire were studied: age, sex, ethnicity, parity, mode of delivery, birth gestational week, birth weight (kg), birth height (cm).

### Statistical analysis

The data entries and analysis were computed utilizing the Statistical Package for the EpiData 3.0 and SPSS 26. T tests and chi-square tests were used to compare the mean levels of continuous variables and percentages of categorical variables among children and adolescents with different feeding mode statuses. According to different data types of the independent variables and dependent variables, we choose the following three different regression models to perform multivariate analysis on the data. A multiple linear regression model was used to analyze the relationship between the duration of breastfeeding (as a continuous variable) and BMI. A generalized linear model was used to analyze the relevance between the duration of breastfeeding (as a categorical variable) and BMI. The relationship between the duration of breastfeeding (as a continuous variable and categorical variable) and the prevalence of overweight and obesity was analyzed by logistic regression analysis. The criterion for statistical significance was a *P* value < 0.05.

## Results

### General Characteristics

A total of 10,753 children and adolescents participated in this study, including 5370 boys (49.93%) and 5383 girls (50.07%). Descriptive statistics of the sociodemographic variables are shown in Table 1.

**Table 1:** Relationship between demographic characteristics and breastfeeding of children and adolescents (aged 6 to 16 years) in Shibei District of Qingdao, China

	Duration of breastfeeding (months)				
	< 12 months (n 4573)		≧12 months (n 6180)		
Characteristics	Mean or n	SD or %	Mean or n	SD or %	*P* value
Age^a^	9.45	2.27	9.24	2.22	< 0.001
Sex^b^					
Male	2196	40.89	3174	59.11	0.001
Female	2377	44.16	3006	55.84	
Ethnicity^b^					
Han	4495	42.49	6084	57.51	0.536
Others	78	44.83	96	55.17	
Parity^b^					
First	4047	43.89	5174	56.11	< 0.001
Others	526	34.33	1006	65.67	
Birth method^b^					
Vaginal delivery	1996	39.00	3122	61.00	< 0.001
Cesarean delivery	2577	45.73	3058	54.27	
Gestational age at birth^b^					
Preterm birth	272	54.51	227	45.49	< 0.001
Full-term birth	4100	41.77	5715	58.23	
Post-term birth	201	45.79	238	54.21	
Birth weight (kg)^a^	3.49	0.54	3.52	0.54	0.001
Birth height (cm)^a^	50.89	1.91	50.97	1.83	0.264
BMI (kg/m2)^a^	18.47	4.17	17.91	3.83	0.001
Body type^b^					
Normal	2762	41.71	3860	58.29	0.030
Overweight	795	43.85	1018	56.15	
Obesity	1016	43.83	1302	56.17	

^a^Student’s t-test was used, the number indicate mean and SD

^b^Chi-square(χ2) test was used, the number indicate percentage value

### Relationship between the duration of breastfeeding and BMI

After adjusting for covariates, there was a significant negative correlation between breastfeeding duration and BMI (β = -0.025, 95% CI: -0.033, -0.005, *P* < 0.01). After classifying the subjects into two groups according to their sex, it was also discovered that a negative correlation still existed between boys (β = -0.040, 95% CI: -0.052, -0.012, *P* < 0.01) and obese girls (β = -0.105, 95% CI: -0.141, -0.034, *P* < 0.01). The BMI of those who were breastfed for more than 12 months was significantly lower than that of those who were breastfed for less than 12 months (β = -0.274, 95% CI: -0.422, -0.127, *P* < 0.01) (Table 2).

**Table 2:** Relationship between breastfeeding and BMI (kg/m^2^) of children and adolescents (aged 6 to 16 years) in ShiBei District of Qingdao, China

Population	Breastfeeding		All weight types		Overweight		Obesity	
			Crude β(95%CI)	Adjusted β(95%CI)	Crude β(95%CI)	Adjusted β(95%CI)	Crude β(95%CI)	Adjusted β(95%CI)
All	Continuous^a^		0.025 (-0.034, -0.006)**	-0.025 (-0.033, -0.005)**	-0.033 (-0.035,0.006)	-0.038 (-0.037,0.004)	-0.088 (-0.097,-0.035)**	-0.091 (-0.100, -0.038)**
	Categorical ^b^	< 12 months	ref	ref	ref	ref	ref	ref
		≧12momths	-0.284 (-0.432, -0.137)**	-0.274 (-0.422, -0.127)**	-0.172 (-0.380,0.035)	-0.200 (-0.409,0.008)	-0.419 (-0.740, -0.099)**	-0.435 (-0.757, -0.114)**
In boys	Continuous ^a^		-0.040 (-0.052, -0.012)**	-0.040 (-0.052, -0.012)**	-0.076 (-0.057, -0.006)**	-0.084 (-0.061, -0.009)**	-0.081 (-0.092, -0.020)*	-0.082 (-0.094,-0.020)*
	Categorical ^b^	< 12 months	ref	ref	ref	ref	ref	ref
		≧12momths	-0.441 (-0.657, -0.225)**	-0.440 (-0.655, -0.224)**	-0.372 (-0.639, -0.104)**	-0.415 (-0.684, -0.146)**	-0.445 (‘-0.830, -0.061)*	-0.451 (-0.836, -0.066)*
In girls	Continuous ^a^		-0.017 (‘-0.032,0.007)	-0.017 (‘-0.032,0.007)	0.014 (-0.026,0.040)	0.010 (-0.028,0.038)	-0.098 (-0.136, -0.029)**	-0.105 (-0.141, -0.034)**
	Categorical ^b^	< 12 months	ref	ref	ref	ref	ref	ref
		≧12momths	-0.196 (-0.394,0.001)	-0.181 (-0.379,0.018)	0.030 (-0.294,0.354)	0.020 (-0.304,0.345)	-0.402 (-0.953,0.149)	-0.426 (‘-0.981,0.130)

*BMI* Body Mass Index, *CI* confidence interval

^a^Multiple liner regression was used to analyze the relationship between breastfeeding duration (as a continuous variable) and BMI

^b^Generalized liner model was used to analyze the relationship between breastfeeding duration (as a categorical variable) and prevalence of overweight and obesity; All the models were adjusted for age, sex, ethnicity, parity, birth method, gestational age at birth, birth weight (kg), birth height (cm)

*P < 0.05

**P < 0.01

### Relationship between the duration of breastfeeding and the prevalence of overweight and obesity

Among boys, the longer the breastfeeding duration was, the lower the prevalence of obesity was (OR = 0.987, 95% CI: 0.974,0.999, *P *< 0.05) in our study, after making adjusting for covariates. The prevalence of obesity among participants whose breastfeeding durations were more than 12 months was significantly lower (OR = 0.853, 95% CI: 0.748,0.974, *P* < 0.05) (Table 3). Among boys aged 9 to 11 years, a significant negative relationship between the duration of breastfeeding and the prevalence of obesity was found (OR = 0.978, 95% CI: 0.958,0.999, *P* < 0.05), and the prevalence of obesity was lower in the participants who were breastfed for more than 12 months (OR = 0.787, 95% CI: 0.653,0.975, *P* < 0.05) (Table 4).

**Table 3:** Relationship between breastfeeding and the prevalence of overweight and obesity among children and adolescents (aged 6 to 16 years) in Shibei District of Qingdao, China

Population	Breastfeeding		Prevalence of overweight			Prevalence of obesity		
			n	Crude OR (95%CI)	Adjusted OR (95%CI)	n	Crude OR (95%CI)	Adjusted OR (95%CI)
All	Continuous		8365	0.996(0.986,1.006)	0.996(0.986,1.006)	8940	0.995(0.986,1.005)	0.998(0.988,1.007)
	Categorical	< 12 months	3487	ref	ref	3778	ref	ref
		≧12momths	4878	0.916(0.825,1.018)	0.918(0.826,1.021)	5162	0.917(0.834,1.009)	0.923(0.838,1.018)
In boys	Continuous		3990	0.992(0.978,1.006)	0.992(0.978,1.006)	4340	0.986(0.974,0.998)*	0.987(0.974,0.999)*
	Categorical	< 12 months	1599	ref	ref	1766	ref	ref
		≧12momths	2391	0.911(0.789,1.052)	0.932(0.805,1.078)	2574	0.856(0.752,0.974)*	0.853(0.748,0.974)*

Logistic regression analysis was used to analyze the relationship between breastfeeding and prevalence of overweight and obesity. Adjusted for age, sex, ethnicity, parity, birth method, gestational age at birth, birth weight (kg), birth height (cm)

*CI* confidence interval, *OR* odds ratio

^*^P < 0.05

**Table 4:** Relationship between duration of breastfeeding and the prevalence of overweight and obesity amnog boys (aged 6 to 16 years) in Shibei District of Qingdao, China

			Prevalence of overweight			Prevalence of obesity		
Age	Breastfeeding		n	Crude	Adjusted	n	Crude	Adjusted
				OR (95%CI)	OR (95%CI)		OR (95%CI)	OR (95%CI)
6–8 years	Continuous		419	0.991(0.971,1.012)	0.990(0.970,1.011)	675	0.991(0.973,1.008)	0.98(0.971,1.007)
	Categorical	< 12 months	160	ref	ref	263	ref	ref
		≧12momths	259	0.953(0.761,1.194)	0.948(0.756,1.189)	412	0.923(0.764,1.114)	0.89(0.740,1.085)
9–11 years	Continuous		398	0.991(0.969,1.014)	0.993(0.970,1.016)	525	0.974(0.954,0.994)*	0.97(0.958,0.999)*
	Categorical	< 12 months	172	ref	ref	248	ref	ref
		≧12momths	226	0.868(0.688,1.095)	0.875(0.691,1.108)	277	0.738(0.598,0.910)*	0.787(0.653,0.975)*
≧12 years	Continuous		213	1.007(0.973,1.041)	1.009(0.974,1.044)	180	0.997(0.962,1.033)	0.999(0.964,1.035)
	Categorical	< 12 months	98	ref	ref	86	ref	ref
		≧12momths	115	1.006(0.727,1.392)	1.009(0.727,1.400)	94	0.937(0.664,1.322)	0.921(0.650,1.306)

Logistic regression analysis was used to analyze the relationship between breastfeeding duration and prevalence of overweight and obesity. Adjusted for age, sex, ethnicity, parity, birth method, gestational age at birth, birth weight (kg), birth height (cm)

*CI* confidence interval, *OR* odds ratio

^*^P < 0.05

## Discussion

Overweight and obesity among children and adolescents have long been a focus of public concern. It is well-known that overweight and obesity are affected by many factors, such as genetic factors, socioeconomic factors, educational level, diet habits, and so on [19, 20]. In this study, the related factors included age, gender, parity, delivery mode, birth weight, birth length and breastfeeding (Supplementary Table 1). We found that students aged 6 to 11 years had a higher risk of being overweight and obesity than students aged more than 12 years. Meanwhile, boys were more likely to suffer overweight and obesity than girls. The possible reason for the gender differences is that boys consumed more fast food and spend more time in sedentary activities, including watching and using computers, phones and tablets [21, 22]. We also found the prevalence of overweight and obesity in students delivered by cesarean section were higher. A recent study has shown cesarean section could affect the colonization pattern of intestinal bacteria in infants, which might lead to overweight and obesity in children and adolescents [23]. First born was one risk factor for overweight and obesity in our study, although the reasons weren't clear. It has been widely proved that the physical development situation of infants has long-term effect on children's physical growth, which even persists into their adolescence [24, 25]. Breast milk is the main food source in infancy, which is closely related to infant nutrition and physical development [26, 27]. But the research on breastfeeding and overweight or obesity among children and adolescents is inadequate [27, 28]. Some new findings which differ from previous studies have been obtained in this study.

The WHO and United Nations International Children's Emergency Fund (UNICEF) recommend that breastfeeding be initiated within one hour of birth, that it continues with no other foods or liquids for the first six months of life, and that it be continued with complementary feeding until at least 24 months of age. Globally, the overall rate of EBF for infants under six months old was 40% in 2017 [29]. However, in China only 29.2% of infants under 6 months were breastfed exclusively in 2017 [30]. The problem of low breastfeeding rates is even more serious in the United States. For example, in 2015, the mean prevalence of EBF in children until the age of six months was 24.9% in the United States [31]. Currently, for 89.8% of the children and adolescents in the Shibei District of Qingdao, the breastfeeding duration was more than 6 months and 57.5% of the children and adolescents’ breastfeeding durations were more than 12 months, which is significantly higher than the overall level worldwide. The decision of how long breastfeeding lasts is strongly influenced by economic, environmental, social, and political factors, such as inadequate health care support, the marketing of baby foods, and workplace support for women [29]. In addition, our study indicated that infants who are not the first-born child or those who are delivered vaginally are more likely to breastfed for more than 12 months. A similar conclusion was reached in Du Li et al.’s study [32]. This may have to do with the fact that infants delivered vaginally are more likely to stimulate breast milk secretion, and that mothers with two or more children are more aware of the advantages of breastfeeding.

To date, most studies have confirmed that prolonging the duration of breastfeeding in infancy can reduce the prevalence of overweight and obesity in children and adolescents. For example, studies conducted in Hong Kong [33] and Spain [34] suggested that the BMI of formula-fed infants increased faster than that of breastfed infants. Spain’s birth cohort study also found that babies in the region who had never been breastfed will had a 7.8% increased risk of childhood obesity and overweight, and that every week of breastfeeding reduced their childhood BMI by 3.5% [34]. Our study proved that the longer the breastfeeding duration was during infancy, the lower the BMI would be in childhood and adolescence. However, in a cohort study involving 5-year-old Swedish children, Huus et al. found no relationship between EBF and the risk of overweight including obesity (OR = 1.22; 95% CI: 0.81-1.83) [35]. Durmus et al. also reported similar findings (OR = 1.20; 95% CI: 0.98-1.47) in Dutch children at the age of 3 [14]. These inconsistences in the results of different studies may be attributed to the children’s age, parental country of birth, parental age, parental smoking status, education level and cultural differences [19]. The related biological mechanism of obesity in children may be closely related to protein intake and energy metabolism. First, studies have confirmed that breast milk itself contains hormones that regulate energy metabolism and food intake, such as leptin and adiponectin. Breastfeeding can regulate the feeding centre and help to establish a dietary habit of consuming energy on demand in infancy, thereby reducing the occurrence of overeating [36]. Second, the protein and calorie content of breastmilk is significantly lower than that of formula milk powder, which can prevent the excessive secretion of insulin and insulin-like growth factors, thereby reducing the deposition of fat and the increase of fat cells [37]. Third, breastfeeding can provide enough nutrition for the infant so that solid foods do not need to be introduced until he or she is 6 months old. The early introduction of solid foods has also been linked to higher risks of obesity [38]. Finally, recent studies suggest that breastfed infants are more likely to accept the low-calorie complementary foods such as vegetables, and are more inclined to form a low-calorie diet in later stages of life, thus reducing the total dietary calorie intake of children and adolescents [39].

In recently published articles, a consensus has not been reached about the duration at which breastfeeding significantly reduces the incidence of overweight and obesity in children. For example, Fallahzadeh et al. found that breastfeeding for more than 24 months was a protective factor for children from becoming overweight [40]. Australian researchers conducted a survey with 2868 infants and found that children who breastfed for less than 4 months had a significantly increased risk of childhood weight exceeding the 95^th^ percentile of children of the same age and sex [41]. Research in Croatia indicated that breastfeeding for more than 6 months was a protective factor for overweight and obesity in children aged 6 to 11 years [42]. However, our study considered that infants who were breastfed for more than 12 months were significantly less likely to be overweight and obese in their childhood and adolescent years. At present, the reasons for the differences in the optimal duration of breastfeeding in different studies are still unclear.

In addition, we discovered that prolonged breastfeeding could reduce the prevalence of childhood obesity, especially boys between the ages of 9 and 11 years. In a longitudinal study with 1,037 children, Poulton and Williams also pointed out that the protective effects of breastfeeding on childhood overweight were relatively weak up to the age of 7 years and then strengthened in late childhood (from the ages of 9 to 11 years) [43]. Meanwhile, German logistic regression analyses that classified subjects into age groups, also indicated that the protective effects of breastfeeding were the most significant from the ages of 7 to10 years [44]. The reason why this correlation is more pronounced in boys is currently unclear but there are several possible reasons. First, due to girls’ intrinsically high insulin resistance, girls have been found to have higher levels of triglycerides and lower concentrations of high-density lipoprotein than boys of the same age, which indicates that metabolic disturbances are more advanced in girls than in boys [45]. Second, this correlation be affected by factors such as family education and social culture. At the ages of 9 to 11 years, girls’ self-awareness begins to mature gradually, and they tend to pay attention to their own image and consciously avoid obesity. However, Chinese culture has few restrictions on the body shapes of boys [46]. Third, epidemiological studies have shown that the prevalence of overweight and obesity in boys is higher than that in girls [19, 20], and this study also had the same findings. This may make it easier to find the relationship between overweight in boys obesity and breastfeeding.

There are several strengths in this study. First, this study was carried out in the urban area of an emerging city in China, where there is currently a lack of research on the relationship between breastfeeding and obesity and overweight. Second, among previous studies, some only involved children, and some only focused on adolescents. Our subjects included both children and adolescents aged from 6 to 16 years. Surely, there are some limitations in the present study. First, the participants in this study were only from the Shibei District of Qingdao, which may have led to selection bias. However, the sample size of this study was large, and it is still representative to a certain extent. Second, some of the data in this study were collected through questionnaires, which are prone to recall bias. We excluded the data with missing variables, which reduced the recall bias to a certain extent. Third, Although we tried our best to identify relevant factors that may affect overweight and obesity, our data are still far from perfect, which may change the results from adjusting to potential confounding. Our future research will focus on these covariates.

## Conclusions

Breastfeeding can significantly reduce the prevalence of overweight and obesity among children and adolescents aged 6 to 16 years. Our results will improve understanding regarding the health benefits of breastfeeding in children and adolescents. Meanwhile, in this study, students who were breastfed for more than 12 months had a lower risk of developing overweight and obesity, especially boys between the ages of 9 and 11. It could contribute in the establishment of breastfeeding guidelines to improve the global problem of childhood obesity.

## Supplementary Information

**Additional file 1: Supplementary Table 1. **Relationship between demographiccharacteristics and body types of children and adolescents (aged 6 to 16 years)in Shibei District of Qingdao, China.

## Data Availability

The datasets collected and analyzed during this study are not publicly available due to the Qingdao Municipal Center for Disease Control and Prevention policy but are available from the corresponding author on reasonable request.

## Abbreviations

BMI: Body Mass Index
EBF: Exclusive Breastfeeding
WHO: World Health Organization
UNICEF: United Nations International Children's Emergency Fund
