# Supplementary material for: Breastfeeding and overweight/obesity among children and adolescents: a cross-sectional study
Source: BMC Pediatr. 2022 Jun 16;22:347. doi: 10.1186/s12887-022-03394-z (PMC9202207; doi:10.1186/s12887-022-03394-z)
Supplement: Supplementary file 1 — Additional file 1: Supplementary Table 1. Relationship between demographiccharacteristics and body types of children and adolescents (aged 6 to 16 years)in Shibei District of Qingdao, China. [file 12887_2022_3394_MOESM1_ESM.docx]

**Supplemental Material**

**Breastfeeding and** **Overweight/Obesity** **among Children and Adolescents: A Cross-Sectional Study**

Fange Liu ^1,*,a^, Di Lv^2,3,a^ , Lumin Wang ^4^, Xiaoyu Feng ^1^, Rongjun Zhang ^1^, Wendong Liu ^2^ , Wenchao Han^2^

^1^ Pediatric Nephrology and Endocrinology Department, the First Affiliated Hospital of Shandong First Medical University &Shandong Provincial Qianfoshan Hospital, Jinan 250014, China;

^2^ Department of Pediatrics, Qingdao Municipal Hospital, Affiliated to Qingdao University, Qingdao 266071, China

^3^ Qingdao Medical College, Qingdao University, Qingdao 266071, China

^4^Department of Pediatrics, Qilu Hospital of Shandong University, Qingdao, Shandong 266035，China

**Contents**

**Supplementary** **[Table 1.](#_Toc1806_)** [Relationship between demographic characteristics and body types of children and adolescents (aged 6 to 16 years) in Shibei District of Qingdao, China.](#_Toc1806_)

**Supplementary Table 1**

| Relationship between demographic characteristics and body types of children and adolescents (aged 6 to 16 years) in Shibei District of Qingdao, China. | | | | | | | |
| --- | --- | --- | --- | --- | --- | --- | --- |
|  | Body types | | | | | | |
|  | normal (n 6622) | |  | overweight/obesity (n 4131) | |  |  |
| Characteristics | Mean or n | SD or % |  | Mean or n | SD or % |  | P value |
| Age(years)^a^ | 9.40 | 2.26 |  | 9.20 | 2.21 |  | <0.001 |
| 6-8^b^ | 2995 | 60.06 |  | 1992 | 39.94 |  | <0.001 |
| 9-11^b^ | 2452 | 61.93 |  | 1507 | 38.07 |  |  |
| ≧12^b^ | 1175 | 65.02 |  | 632 | 34.98 |  |  |
| Sex^b^ |  |  |  |  |  |  |  |
| Male | 2960 | 55.12 |  | 2410 | 44.88 |  | <0.001 |
| Female | 3662 | 68.03 |  | 1721 | 31.97 |  |  |
| Ethnicity^b^ |  |  |  |  |  |  |  |
| Han | 6505 | 61.49 |  | 4074 | 38.51 |  | 0.122 |
| Others | 117 | 67.24 |  | 57 | 32.67 |  |  |
| Parity^b^ |  |  |  |  |  |  |  |
| First | 5623 | 60.98 |  | 3598 | 38.52 |  | 0.002 |
| Others | 999 | 65.21 |  | 533 | 34.79 |  |  |
| Birth method^b^ |  |  |  |  |  |  |  |
| Vaginal delivery | 3321 | 64.89 |  | 1797 | 35.11 |  | <0.001 |
| Cesarean delivery | 3301 | 58.58 |  | 2334 | 41.42 |  |  |
| Gestational age at birth^b^ |  |  |  |  |  |  |  |
| Preterm birth | 288 | 57.72 |  | 211 | 42.28 |  | 0.125 |
| Full-term birth | 6072 | 61.86 |  | 3743 | 38.14 |  |  |
| Post-term birth | 262 | 59.68 |  | 177 | 40.32 |  |  |
| breastfeeding^b^ |  |  |  |  |  |  |  |
| No | 92 | 58.60 |  | 65 | 41.40 |  | 0.439 |
| Yes | 6530 | 61.63 |  | 4066 | 38.37 |  |  |
| Duration of breastfeeding (months)^b^ |  |  |  |  |  |  |  |
| <12 months | 2762 | 60.40 |  | 1811 | 39.60 |  | 0.030 |
| ≧12months | 3860 | 62.46 |  | 2320 | 37.54 |  |  |
| Birth weight (kg)^a^ | 3.48 | 0.54 |  | 3.55 | 0.54 |  | <0.001 |
| Birth length (cm)^a^ | 50.84 | 1.85 |  | 51.09 | 1.88 |  | <0.001 |
| BMI (kg/m2)^a^ | 16.52 | 1.77 |  | 22.11 | 3.82 |  | <0.001 |
| Note: a, Student’s t-test was used, the number indicate mean and SD; b, Chi-square(χ2) test was used, the number indicate percentage value. | | | | | | | |
